# Reconstruction of membrane current by deconvolution and its application to membrane capacitance measurements in cardiac myocytes

**DOI:** 10.1371/journal.pone.0188452

**Published:** 2017-11-22

**Authors:** Matej Hoťka, Ivan Zahradník

**Affiliations:** 1 Department of Muscle Cell Research, Institute of Molecular Physiology and Genetics, Centre of Biosciences, Slovak Academy of Sciences, Bratislava, Slovak Republic; 2 Department of Biophysics, Faculty of Science, Pavol Jozef Šafárik University, Košice, Slovak Republic; University of Minnesota, UNITED STATES

## Abstract

Correct detection of membrane currents under whole-cell patch–clamp conditions is limited by the transfer function of a recording system. The low-pass output filter of a recording amplifier alters the time course of membrane current and causes errors in relevant descriptors. To solve these problems, we developed a practical procedure for reconstruction of the time course of membrane currents based on deconvolution of recorded currents in frequency domain. The procedure was tested on membrane capacitance estimates from current responses to step voltage pulses. The reconstructed current responses, in contrast to original current records, could be described exactly by an adequate impedance model of a recorded cell. The reconstruction allowed to increase the accuracy and reliability of membrane capacitance measurements in wide range of cell sizes and to suppress the cross-talk errors well below the noise. Moreover, it allowed resolving the instabilities in recording conditions arising from parasitic capacitance and seal resistance variation. Complex tests on hardware models, on simulated data sets, and on living cells confirmed robustness and reliability of the deconvolution procedure. The aptitude of the method was demonstrated in isolated rat cardiac myocytes by recording of spontaneous vesicular events, by discerning the formation of a fusion pore, and by revealing artefacts due to unstable seal resistance.

## Introduction

Electrical phenomena originating at the cell surface can be used to characterize mechanisms of related cellular functions down to the single molecule level. Their measurement is limited by the bandwidth and resolution of the recording method that define the information content of the measured signal. The patch–clamp technique and design of recording amplifiers [1, 2] allowed high-resolution measurements of both the passive and active electrical characteristics in most cells types. A change of voltage across the cell membrane causes a current response proportional to its impedance and activity of ion channels and transporters, which vary in a characteristic way for a given physiological state of recorded cell and experimental conditions. In practise, the bandwidth of a measurement is limited by the transfer function of the whole recording system. Commercial recording amplifiers are well compensated up to 100 kHz bandwidth with the transfer function close to one [2]. In practise, the overall transfer function is shaped by impedance of the cell and of the recording patch electrode at the input of recording amplifier, and by the low-pass filter at the output of the amplifier. The resulting transfer function is not ideal and makes recording of dynamic phenomena at high accuracy and resolution problematic. The capacitance of the patch pipette and its holder should be compensated at the input to keep the amplifier stable, since it generates fast and large charging current proportional to the rate and amplitude of command voltage [2]. The uncompensated charging current will be transferred by the amplifier and convolved with the cell membrane current by the output filter, used for suppression of the high-frequency noise and for correct digitization. Moreover, it also distorts the input current and introduces artefacts not present at the input. In the case of the whole-cell membrane current recording, for instance, the current response to a step voltage stimulus should be an exponentially decreasing signal. However, the output filter transforms it into a two phasic response that deviates at early times substantially from expected exponential decrease. Moreover, due to the Gibb’s phenomenon [3] the filter adds damped oscillations proportional to the input signal. These effects change the information content of high frequency signals that defines kinetics of fast processes. The problem is relevant to measurements based on fast changes of membrane potential even when the voltage-clamp is fast, for instance, activation of gating currents, activation of very fast ionic currents, deactivation of ionic currents (tail or instantaneous currents), or high resolution recording of membrane capacitance.

Current responses to square voltage pulse are used for evaluation of the cell membrane capacitance and conductance. According to the theory, the current response should be described by an exponential-like decay characterized by the two parameters and the access resistance of the recording patch pipette [4]. To avoid interfering distortions in current responses, only a less affected part of the recorded current, i.e., with the first 60 μs omitted, was fitted by appropriate equations that need the peak current amplitude value [4]. Exact estimation of the peak current amplitude needs extrapolation over the omitted 60 μs to time of voltage change. This requires proper adjustment of the zero time of the recorded current relative to the zero time of the step voltage onset that compensates delay introduced by the filters in current and voltage paths [4]. Otherwise, the error in peak current amplitude passes to the estimate of the access resistance value and subsequent calculations of membrane capacitance and membrane resistance. In practice, the zero time had to be adjusted by systematic variation until correlated changes in the evaluated membrane capacitance and membrane resistance data traces were minimal. Thus, although the omission of the first data points reduces the sensitivity of the peak current estimate to changes in pipette capacitance, it reduces the resolution of the membrane capacitance estimate [5]. The reduced resolution could be partially compensated by higher stimulation frequency [6], where possible. For cells of capacitance larger than 10 pF, optimal resolution of membrane capacitance was obtained at stimulation periods of 12 time constants of current relaxation, that is, 6 time constants per voltage change [5].

The increase in computational power of computers gave the square-wave methods [5, 7] potential to outperform sine-wave methods in cell impedance measurements [8, 9,10], due to the relatively simple implementation, higher information content [5], and low sensitivity to membrane resistance changes [11, 5]. However, in fast measurements with a time constant below 200 μs, typical for cells of less than 40 μm in diameter, where the sine-wave methods excel, the square-wave methods start to suffer from errors instigated by an increased contribution of the parasitic capacitance, and by the low-pass antialiasing filter [5]. Consequently, the square-wave method had to be optimized for specific cells and experiments [5]; otherwise, the records of membrane capacitance might contain artefacts presenting as capacitance variances of unknown origin. Still other artefacts may arise from instability in the recorded circuit, especially in the access resistance, but also in the membrane and/or seal resistance. Altogether, these artefacts downgrade reliability of the square wave methods for membrane capacitance measurements.

Generally, understanding of membrane processes would benefit from improvements in fidelity of membrane current recording. Fortunately, according the theory [3], if the transfer function is known it could be used to reconstruct the input current through deconvolution in frequency domain.

In this study, we developed a method for reconstruction of membrane current responses recorded by the whole-cell patch-clamp technique. The essence of the method is a deconvolution procedure that uses experimentally estimated transfer function of the recording system. The method was rigorously tested by hardware and software models. We show that the deconvolution procedure cancels distortions of the membrane current responses caused by low-pass filtering and improves the bandwidth of measurements. Application of the deconvolution method to high-resolution capacitance measurements eliminated cross-talk errors and allowed for the detection of membrane capacitance activity in small postnatal as well as large adult cardiac myocytes. Moreover, it allowed the difficult problems of parasitic capacitance interference and of the seal resistance instability to be tackled.

## Materials and methods

### Patch-clamp experiments

Experimental setup was built around inverted microscope (Diaphot TMD, Nikon, Japan) placed on anti-vibration base (Micro40 M6, Halcyonics, Germany) and shielded by a Faraday cage. Cells were recorded in a 0.5 ml open air chamber with cover glass bottom (Warner Instruments, LLC, USA) and grounded with Ag/AgCl pellet electrode (Warner Instruments). Membrane current was recorded with a patch-clamp amplifier (Axopatch 200B, Axon Instruments Inc., USA) using a glass micropipette fixed to a standard holder of the head-stage preamplifier (CV-203BU, Axon Instruments Inc., USA) mounted on a 4-axis electrical micromanipulator MX 7600R (Siskiyou, USA). Patch pipettes were pulled from borosilicate glass capillaries (Sutter Instruments Co., USA) on Flaming/Brown Micropipette Puller Model P-97 (Sutter Instruments Co., USA). The pipette resistance was typically 2–4 MΩ. Pipette tips were coated with SYLGARD184 (Dow Corning, USA) to reduce stray pipette capacitance and its fluctuations. Formation of the seal between the patch pipette and the cell membrane was monitored by the pipette potential in cell-attached current-clamp mode [12]. A cell was accepted for measurements if the potential stabilized below -40 mV and it withstood rupturing of the membrane patch. The final seal resistance was typically several tens of GΩs.

Ventricular myocytes of rat myocardia were isolated enzymatically [13] from young adult male or neonatal hearts of Han-Wistar rats (Dobra Voda, Slovak Republic). All anaesthetic and surgical procedures were carried out in accordance with the European directive 2010/63/EU, and were approved by the State Veterinary and Food Administration of the Slovak Republic (Ro-2821/09-221, Ro-354/16-221) and by the Ethical Committee of the Institute of Molecular Physiology and Genetics, Slovak Academy of Sciences.

Experiments on isolated cardiac myocytes were performed at room temperature of about 23°C. The standard bath solution contained (in mmol/l): 135 NaCl, 5.4 CsCl, 1 CaCl_2_, 5 MgCl_2_, 0.33 NaH_2_PO_4_, 10 HEPES, 0.01 TTX (pH 7.3; 300 mOsm). The composition of the pipette solution was (in mmol/l): 135 CsCH_3_SO_3_, 10 CsCl, 1 EGTA, 3 MgSO_4_, 3 Na_2_ATP, 0.05 cAMP, 10 HEPES (pH 7.1; 300 mOsm). Osmolarity of all solutions was verified using Osmomat 010 (Gonotec GmbH, Berlin, Germany).

### Impedance measurements and analysis

Whole cell impedance measurements were made using the patch-clamp amplifier in the whole-cell voltage clamp mode with a feedback resistor of 50 MΩ. The gain was set to 0.2 mV/pA to avoid amplifier saturation. The output current was low-pass filtered by the built-in 4-pole Bessel filter set to 10 kHz, digitized at 100 kHz by a 16-bit data acquisition system (Digidata 1320A, Axon Instruments Inc.) and stored in proprietary Axon Binary Files (.abf) for off-line analysis.

The bipolar square-waves voltage stimuli were generated by 16-bit D/A converter (Digidata 1320A). The stimulation period *T*_*S*_ was set in relation to the time constant *τ* of measurement as *T*_*S*_ = 12*τ* (6*τ* for each half-period [5]) and ranged from 20 to 0.6 ms (corresponding to stimulation frequency of 50–1,670 Hz). During impedance measurements, the holding potential was set to 0 mV. Experiments were controlled by software (pClamp 9, Axon Instruments Inc.).

The recorded current responses to square-wave voltage stimuli were processed by the deconvolution procedure specified in the Results. Where indicated, the standard processing method commonly used in patch-clamp laboratories (e.g., pClamp v.10, Molecular Devices, https://mdc.custhelp.com/euf/assets/content/pCLAMP10_User_Guide.pdf) was applied. We followed original procedures [5, 4], in which the first 60 μs of a current record were disregarded and the remaining current was fitted by Eq 1. The peak current amplitude was determined by extrapolation of the best fit to the current response to the new zero time corrected for filter delay. When such estimate of the peak current amplitude was used for calculation of the access resistance by Eq 2A, the estimates of membrane capacitance *C*_*M*_ strongly correlated with *R*_*A*_. The cross-correlation could be minimized, as suggested by Thompson et al. [5], by tuning the zero time correction to a value that provided acceptable cross-correlation in *C*_*M*_ traces. At 10 μs sampling interval, the exact zero time correction required its tuning down to a fraction of microsecond in about 5–10 steps. The cross-correlation error arose due to the improper best fit; the errorneous estimates of the τ and *R*_*A*_ led to the zero time being wrongly projected.

**Fig 1:**
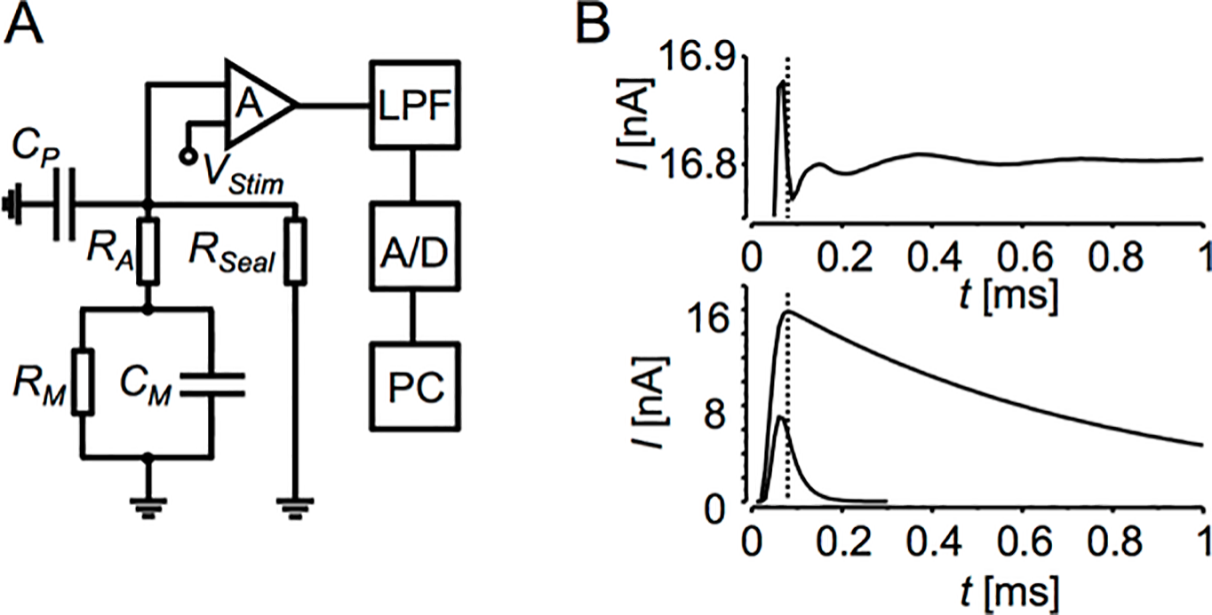
Membrane current recording. **A**: The equivalent circuit of the whole-cell patch-clamp recording configuration. *C*_*M*_—membrane capacitance. *R*_*M*_—membrane resistance. *R*_*A*_—access resistance. *R*_*Seal*_—resistance of the seal between the recording microelectrode and the cell membrane. *C*_*P*_—parasitic capacitance of the recording microelectrode. *V*_*Stim*_—stimulation voltage applied to the measured circuit. A—patch-clamp amplifier. LPF—low-pass filter. A/D—digitizer. PC - computer. **B**: Distortions in current responses due to the low-pass filtering. Upper panel–a detail of the recorded current response of 4.7431 MΩ resistor to 80 mV step low-pass filtered by 10 kHz 4-pole Bessel filter and sampled at 100 kHz. The parasitic capacitance was compensated. Averaging was used to eliminate the noise. The time to half-maximal amplitude was 33.8 μs. Lower panel–current responses of a large (*C*_*M*_ = 160 pF) and a small (*C*_*M*_ = 5 pF) hardware cell recorded with the same *R*_*A*_ = 4.7431 MΩ and filtered and sampled as in upper panel. The dotted lines in both panels indicate the border between the disregarded and fitted parts of the recorded current responses.

The parameters of current responses were estimated by fitting Eq 1 to both half-periods of current responses *I*_*M*_*(t)* and averaged.

$$I_{M}\left(t\right)=I_{S}+I_{P}\cdot e^{\left(\frac{-t}{\tau }\right)},$$ (1)

where *I*_*P*_ is the peak current, *I*_*S*_ is the steady current, and *τ* is the time constant. The parameters of the recorded cell circuit, approximated by membrane resistance *R*_*M*_, membrane capacitance *C*_*M*_, and access resistance *R*_*A*_ (Fig 1) were calculated from the estimated parameters of current responses (*I*_*P*_, *I*_*S*_, *τ*) using Eqs 2A–2C.

$$R_{A}=\frac{V_{Stim}}{I_{P}+I_{S}},$$ (2A)

$$R_{M}=\frac{V_{Stim}}{I_{S}}-R_{A},$$ (2B)

$$C_{M}=\tau \cdot \left(\frac{1}{R_{M}}+\frac{1}{R_{A}}\right).$$ (2C)

Notice that Eq 2A contains *I*_*S*_ since in practise the peak current *I*_*P*_ is not calculated from the zero current level, but due to the use of symmetrical bipolar stimulation it is estimated from the steady state current *I*_*S*_ of the preceding half-period. The analysis was performed off-line using routines written in MATLAB (v.2014b, Mathworks Inc., Natick, MA, USA) and implemented in the software MAT-MECAS (http://mat-mecas.sourceforge.net).

Standard deviation *σC*_*M*_ of the analysed time series of capacitance measurements was determined for the bandwidth of interest *B*, typically 50 Hz. Since the measurements were performed at a stimulation frequency *f*_*S*_, the number of periods to be averaged *m* was found using the relationship *m* = *f*_*S*_ */B*. The theoretical limit of *C*_*M*_ resolution for the given bandwidth was calculated using Eq 3 considering white thermal noise [5]:

$$\sigma C_{M}=C_{M}\frac{\sqrt{4kTBR_{A}}}{V_{Stim}},$$ (3)

where *k* is the Boltzmann constant and *T* is the absolute temperature.

### Simulations and evaluation of errors

All simulations were performed in MATLAB (v.2014b, Mathworks).

To test the performance of the deconvolution procedure, we simulated 500 records of membrane current responses using Eqs 4A–4D and a seeding set of random triplets of circuit parameter values generated uniformly within ranges *C*_*M*_ (pF) ∈ [5, 200], *R*_*M*_ (GΩ) ∈ [0.02, 2], *R*_*A*_ (MΩ) ∈ [2, 20]:

$$I(t)=V_{Stim}\left[\frac{1}{R_{A}}\cdot e^{\frac{-t}{\tau }}+\frac{1}{R_{M}+R_{A}}\left(1-e^{\frac{-t}{\tau }}\right)\right],$$ (4A)

where

$$\tau =\frac{C_{M}\cdot R_{M}\cdot R_{A}}{R_{M}+R_{A}},$$ (4B)

and the theoretical values or current parameters are:

$$I_{P}=\frac{V_{Stim}}{R_{A}},$$ (4C)

$$I_{S}=\frac{V_{Stim}}{R_{M}+R_{A}}.$$ (4D)

To simulate real recording conditions, the calculated membrane current responses were digitally filtered with the MATLAB function “filter”. The digital filter was an implementation of the analog 10 kHz 4-pole Bessel filter, using the method of invariance of impulse response “impinvar”, while the analog filter was emulated by the MATLAB function “besself”.

To evaluate the absolute error of estimate of circuit parameters, the simulated, digitized and filtered current responses were analysed in the same way as real records (using Eqs 1 and 2A–2C), and the difference between the seeded and the estimated parameter values was calculated.

For evaluation of the crosstalk error due to R_A_ variation, we simulated two periods of current responses for each random triplet of parameter values, so that the R_A_ value of the second period was increased by 1 MΩ. The simulated current responses were analysed in the same way as real records using Eqs 1 and 2A–2C. Crosstalk errors were evaluated as the difference between the estimated parameters of the two sets of simulated current responses.

To assess the effect of *R*_*A*_ variation on the resolution of membrane capacitance measurements, the current responses were calculated for a cell circuit with *C*_*M*_ = 50 pF, *R*_*M*_ = 200 MΩ, and *R*_*A*_ values fluctuating randomly around the mean of 5 MΩ. The resolution was evaluated as standard deviations *σC*_*M*_ estimated at the corresponding standard deviations *σR*_*A*_ (22.6, 45.2, 90.4, 135.6, 180.8, 361.7, and 452.1 kΩ), at the bandwidth normalized to 50 Hz.

The dependence of the circuit parameter estimates on the resistance *R*_*Seal*_ was simulated using Eq 5.

$$I(t)=V_{Stim}\left[\frac{1}{R_{A}}\cdot e^{\frac{-t}{\tau }}+\frac{1}{R_{M}+R_{A}}\left(1-e^{\frac{-t}{\tau }}\right)+\frac{1}{R_{Seal}}\right],$$ (5)

The Eq 5 differs from Eq 4A by the last term that includes the *R*_*Seal*_. The current responses for a cell with *C*_*M*_ = 140 pF, *R*_*M*_ = 500 MΩ, and *R*_*A*_ = 4 MΩ were stimulated with *V*_*Stim*_ amplitude of ±10 mV and a period of 6*τ*. The seal resistance *R*_*Seal*_ was incremented by 100 MΩ, period by period, from 1 to 150 GΩ.

## Results

Homogenously voltage-clamped cell in the whole-cell patch-clamp recording configuration (Fig 1A) should respond to a square voltage stimulus by a membrane current that complies to Eq 4A. In practice, however, the current response is modified by the transfer function of the signal path (Fig 1B). When a step voltage change is applied to a resistor connected to the amplifier, it should evoke a step current response; however, the low-pass filter in the signal path causes dumped ringing lasting for a few hundred microseconds (Fig 1B, upper panel). Since the amplitude of ringing is the larger the larger is the current step, it is advised to compensate the parasitic capacitance at the input of the recording amplifier [2]. The ringing is present also in current responses recorded with the hardware cell model (Fig 1B, lower panel). Although it is difficult to distinguish on the large and fast changing currents, the ringing may influence the precision of fitting of the current time course by a theoretical function. Ringing becomes increasingly important in smaller cells, since the charging current declines with time constant comparable to ringing period of the low-pass filter (compare the upper and lower panels of Fig 1B). Therefore, the recorded currents should be processed appropriately before analysis to estimate the measured parameters correctly.

### The deconvolution procedure

The recorded current, *I*_*R*_(*t*), represents the convolution of the input current *I*_*M*_(*t*) with the impulse response, *h*(*t*), of the signal path [11]:

$$I_{R}\left(t\right)=I_{M}(t)*h(t).$$ (6)

**Fig 2:**
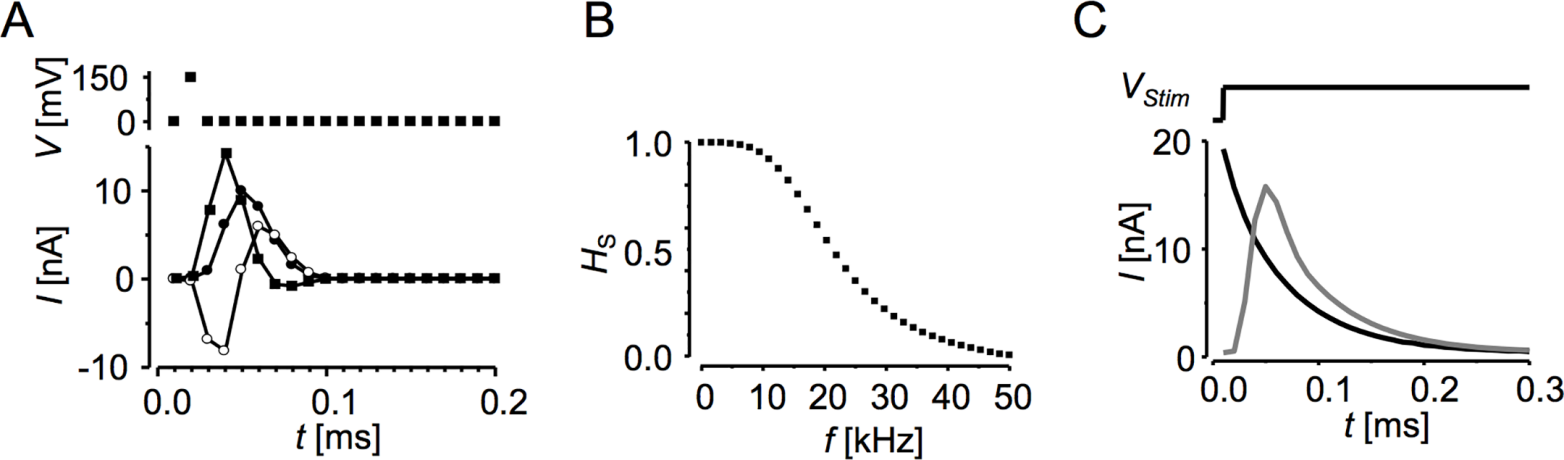
The deconvolution procedure. **A:** Sampled voltage stimulus and current responses (the first 200 microseconds are shown). *Upper panel*: the single sample voltage impulse. *Lower panel*: the sampled current responses obtained by averaging 4096 records to suppress the noise. ●–the averaged current response recorded with the 4.7 MΩ resistor connected between the amplifier input and the ground (R+C response); ○—the averaged current response recorded with the 4.7 MΩ resistor connected to the amplifier input and disconnected from the ground (C-response);. ■—the impulse current response (R-response) obtained by subtracting the C-response from the R+C response. **B:** The characteristic of the signal path in the frequency domain, *H*_*S*_, obtained by FFT of the R-response. **C:** An example of the recorded low-pass filtered current response (grey line) and of the current response reconstructed by deconvolution procedure (black line) obtained with the hardware model cell (*C*_*M*_ = 10 pF, *R*_*M*_ = 500 MΩ, *R*_*A*_ = 4.7 MΩ) to *V*_*Stim*_ = 80 mV. The parasitic capacitance was compensated by the amplifier compensation circuitry.

The original input signal *I*_*M*_(*t*) can be recovered by a deconvolution process, if the impulse response of the recording system is known. For this purpose, we recorded the impulse response of the recording system (Fig 2A). The voltage impulse was represented by a 10-μs 150-mV voltage stimulus (generated by the digitizer set to 100 kHz). When delivered to the amplifier configured with a resistor between the input and the ground, the impulse generated a complex current response (R+C response), which contained the resistive component (R response) combined with the capacitive component (C response) generated by the parasitic capacitance and the inner circuitry of the amplifier. These two capacitive components are always present in the current recording path. Their contribution to the signal should be eliminated to obtain the correct impulse response, *h*(*t*). To this end, the voltage impulse was delivered to the amplifier with the same resistor connected to the input but disconnected from the ground. In this arrangement, the current response contained only the capacitive component (C response). To supress the noise, the R+C and C responses, each of 20 ms duration, were recorded 4096 times and averaged. The average C response was subtracted from the average R+C response and the resulting average R-response was transformed by the fast Fourier transform algorithm (FFT) to the frequency domain and normalized to 0–1 range. This operation yielded the system frequency characteristic *H*_*S*_ in 0.05–50 kHz bandwidth (Fig 2B). A special attention was given to proper grounding of the set-up to eliminate high frequency interference signals, because their presence in the *H*_*S*_ function caused oscillations in the reconstructed currents.

In practice, *H*_*S*_ is applicable to reconstruction of current responses measured with the same sampling and filtering frequency. For best results in cell experiments, the charging current of parasitic capacitance of the recording microelectrode should be properly cancelled either by using the circuitry of the amplifier or by subtraction of pre-recorded capacitive current responses, both in the cell-attached configuration before breaking the patch membrane.

Reconstruction of membrane current responses distorted by the recording path was performed in three steps. First, the recorded current response was transformed to the frequency domain by FFT. Then, the deconvolution was performed in the frequency domain by dividing the recorded current response by *H*_*S*_. Finally, the result was transformed to the time domain by inverse FFT, which yielded the reconstructed current response (Fig 2C).

The reconstructed current responses to square voltage stimuli followed exponential time course and could be approximated by Eq 1. Consequently, the estimated current parameters corresponded to current parameters of the theoretical circuit and Eqs 2A–2C could be used for calculation of the impedance parameters of the recorded circuit.

The whole processing and analysis procedure for deconvolution of membrane current records was written into an interactive software package MAT-MECAS in MATLAB v. 2014b (see: http://mat-mecas.sourceforge.net), which was used also in this study. The software reads records of current responses to square-wave voltages, saved in an appropriate format (the Axon Binary File abf; Axon Instruments Inc., or a text file), and creates records of the time course of *C*_*M*_, *R*_*M*_, *R*_*A*_, and other specified parameters in xlsx format. MAT-MECAS also provides special tools for secondary analyses, including identification of events and artefacts in *C*_*M*_ records. A record of 1000 current responses, each of 100 samples per period, was analysed in about 60 s at full temporal resolution (1 kHz) or in about 3 s if the bandwidth was reduced to 50 Hz. The software application was tested for correct data processing and for correct mathematical processing using simulated data, as these provide exact control of outputs relative to inputs.

### Accuracy and resolution

**Fig 3:**
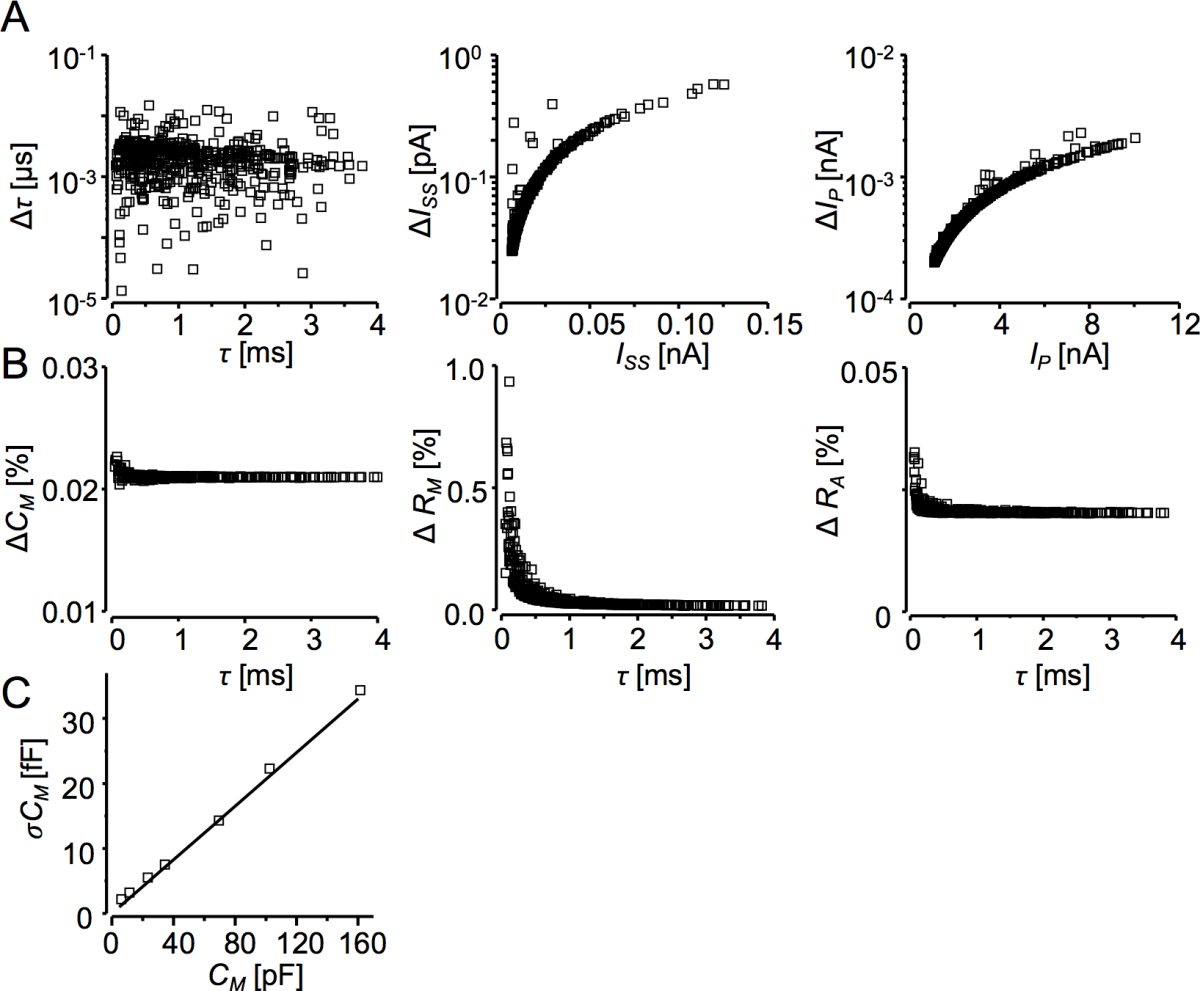
Accuracy and resolution tests of the deconvolution method. **A:** Absolute errors of the estimated parameters of simulated, filtered, and reconstructed current responses (Δ*τ*, Δ*I*_*S*_, Δ*I*_*P*_) plotted against their respective theoretical values. The errors were calculated as differences between the best-fit parameter values of Eq 1 to simulated, filtered, and reconstructed current responses and the theoretical parameter values calculated by Eqs 4B–4D for the set of random triplets of cell parameters (see Materials and Methods). **B:** Relative errors of the circuit parameters estimates (Δ*C*_*M*_, Δ*R*_*M*_, Δ*R*_*A*_), determined from the simulated, filtered, and reconstructed current responses (Eq 4), plotted against calculated *τ* values (Eq 4B) for the respective random triplets of circuit parameters (the same dataset as in **A**). **C**: Standard deviations of membrane capacitance σ*C*_*M*_ of the hardware model cells plotted against *C*_*M*_ values wired in the models (4.7, 10, 22, 33, 68, 101, or 160 pF). In all hardware models, *R*_*A*_ was 4.7 MΩ and *R*_*M*_ was 500 MΩ. Square-wave ±10 mV, 12*τ* periods. The theoretical limit of *C*_*M*_ resolution (black line) was calculated by Eq 3 considering thermal noise. Bandwidth 50 Hz.

The errors arising from the deconvolution procedure itself were assessed in the simulated set of filtered current responses generated with the set of random triplets of circuit parameters, as described in Materials and Methods. The simulated responses were processed by the deconvolution procedure and fitted with Eq 1. Differences between the estimated parameters of current responses and the ones calculated for the given triplet of circuit parameters are plotted in Fig 3A. The deconvolution procedure provided reconstructed currents almost identical to the original ones, since the differences *Δτ*, *ΔI*_*S*_, and *ΔI*_*P*_ were negligible at any combination of the circuit parameter values.

Differences between the circuit parameter values used for simulation and those estimated by the deconvolution procedure are shown in Fig 3B. We plotted the differences as the percentage of the parameter value in the triplet against the corresponding *τ* values calculated for each triplet using Eq 4B. The time constant of current response *τ* is a convenient reference parameter since it incorporates all circuit parameters. It also determines the reliability of the fitting procedure since, due to the constant sampling interval, the number of sampled data points is proportional to *τ*. The relative errors in *C*_*M*_ were under 0.02% and virtually independent of *τ* at any combination of circuit parameter values. The relative error in *R*_*M*_ estimates was less than 0.1% for *τ* > 500 μs and increased to about 1% for *τ* = 50 μs. The relative error in *R*_*A*_ estimates was very low, under 0.03% for *τ* > 100 μs at any combination of parameter values.

The resolution limit of the deconvolution procedure for membrane capacitance measurements was estimated on hardware models made of different capacitors combined with membrane and access resistors (Fig 3C). The deconvolution procedure performed near the physical limit of *C*_*M*_ resolution given by thermal noise (Eq 3), even for very small cells (*C*_*M*_ < 20 pF).

### Cross-talk error

In measurements on real cells, all circuit parameters (Fig 1A) may vary in a rather wide range. Therefore, the parameter cross-talk is a crucial issue of high-resolution measurements. Of special consideration is the access resistance, which solely defines *I*_*P*_, contributes substantially to the time constant of measurement, and may vary by up to several MΩ on short as well as long-time scales. At the same time, the cell membrane resistance *R*_*M*_ may change from a few GΩ to a few tens of MΩ due to experimental interventions or membrane impedance nonlinearities. On the other hand, *R*_*M*_ contributes substantially to the time constant only when comparable to *R*_*A*_, which is not a standard situation.

**Fig 4:**
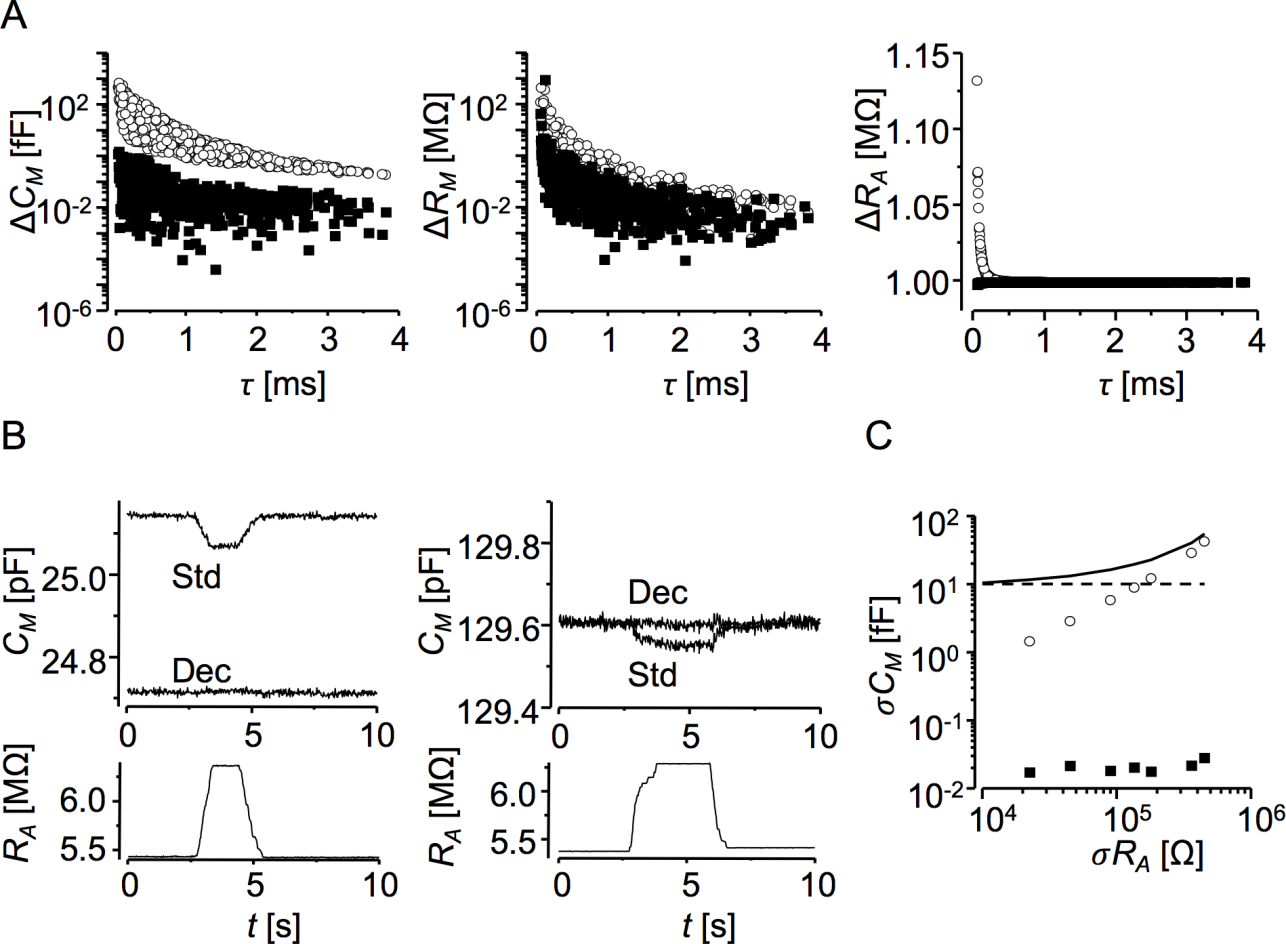
The cross-talk error tests. **A:** Analysis of the simulated, filtered, and reconstructed current records by the deconvolution (■) and the standard procedures (○). The differences in estimates of *C*_*M*_, *R*_*M*_, and *R*_*A*_ before and after increasing the *R*_*A*_ value by 1 MΩ are plotted against the range of pertinent *τ* values (Eq 4B). **B:** Analysis of current records obtained with the use of compensation settings of the recording amplifier. *Upper panels*: time series of *C*_*M*_ estimates. *Lower panels*: time series of *R*_*A*_ estimates. The *R*_*A*_ value was changed manually by about 1 MΩ for 2–3 seconds using the R_A_ compensation knob. The *C*_*M*_ value was set to emulate either a small cell (left panels, *τ* = 136 μs) or a large cell (right panels, *τ* = 713μs). The deconvolution (Dec) and standard (Std) traces were obtained by the deconvolution and standard procedures, respectively. Square-wave ±40 mV, 10 ms periods, bandwidth 50 Hz. **C:** Contribution of the *R*_*A*_/*C*_*M*_ crosstalk error to the overall *C*_*M*_ resolution. A set of filtered current responses was simulated for a cell of *C*_*M*_ = 50 pF, *R*_*M*_ = 200 MΩ, and *R*_*A*_ varying randomly with the same mean of 5 MΩ but different values of standard deviation (500 stimulation periods for each σ*R*_*A*_). The standard deviation σ*C*_*M*_ in the *C*_*M*_ time series was estimated for 50 Hz bandwidth. Dashed line–the thermal noise level calculated by Eq 3. The σ*C*_*M*_ values in the time series of *C*_*M*_ estimates obtained by the deconvolution (■) or standard (○) procedures. Solid line–the sum thermal noise and the standard procedure data. Notably, in the case of the deconvolution procedure (■), the fluctuations in *C*_*M*_ caused by fluctuations of *R*_*A*_ were by 3 orders of magnitude below the thermal noise level.

Propensity of the deconvolution method for cross-talk error was tested by impedance measurements using the same set of parameter triplets as that used for testing the accuracy, to which siblings differing in *R*_*A*_ by 1 MΩ were generated. The Fig 4A shows results of analysis of the simulated current responses related to the time constant of the membrane charging *τ*, through which the interdependence of parameters arises. Except for the very short *τ*’s, the cross-talk of a 1 MΩ change in *R*_*A*_ to measured *C*_*M*_ and *R*_*M*_ was negligible. The differences in *C*_*M*_ estimates were typically much less than 1 fF, while the estimates of *R*_*A*_ changes were almost exactly 1 MΩ (0.9995 MΩ). Analysis of the same data set by the standard procedure (see Methods) returned substantially larger errors in estimates of any impedance parameter (Fig 4A).

The role of cross-talk of *R*_*M*_ variation to other parameters was studied in additional simulations. These revealed that even very large variation of membrane resistance does not present a problem, since the method managed to keep errors of *C*_*M*_ estimation below 0.2 fF when *R*_*M*_ was changed by 100 MΩ in the 0.02–2 GΩ range (not shown).

Additional assessment of the crosstalk error was performed using the built-in compensation circuitry of the recording amplifier [5]. Recordings shown in Fig 4B were made with the input of the amplifier open and with the membrane capacitance compensation set to about 25 or 130 pF to simulate a small or a large cell, respectively. The series resistance compensation was set to about 5.5 MΩ in both cases. During recording, the series resistance compensation was changed to about ~6.5 MΩ and back to emulate fast and large *R*_*A*_ variation. Absence of visible correlated changes in the *C*_*M*_ and *R*_*A*_ traces obtained by the deconvolution procedure, but not in traces obtained by the standard procedure, confirms results of numerical simulations.

In real cell experiments, *R*_*A*_ can vary broadly at various time scales. In the presence of cross-talk, variation of *R*_*A*_ would transpire to *C*_*M*_ recordings, increase the variability of *C*_*M*_ estimates and decrease the resolution of *C*_*M*_ measurements. To assess the effect of cross-talk on *C*_*M*_ resolution, we simulated traces of current responses of a typical cell with *R*_*A*_ fluctuating randomly and compared the standard deviation of *R*_*A*_ with the standard deviation of *C*_*M*_ (Fig 4C). With the use of the deconvolution procedure, variance of *C*_*M*_ due to cross-talk from *R*_*A*_ was three orders below the variance that would result from thermal noise. If the current responses were analysed by the standard procedure, the resulting crosstalk error increased fluctuations of membrane capacitance estimates above the level expected for thermal noise substantially for *R*_*A*_ changes exceeding 20 kΩ, as present in majority of experiments.

### Effect of parasitic capacitance

**Fig 5:**
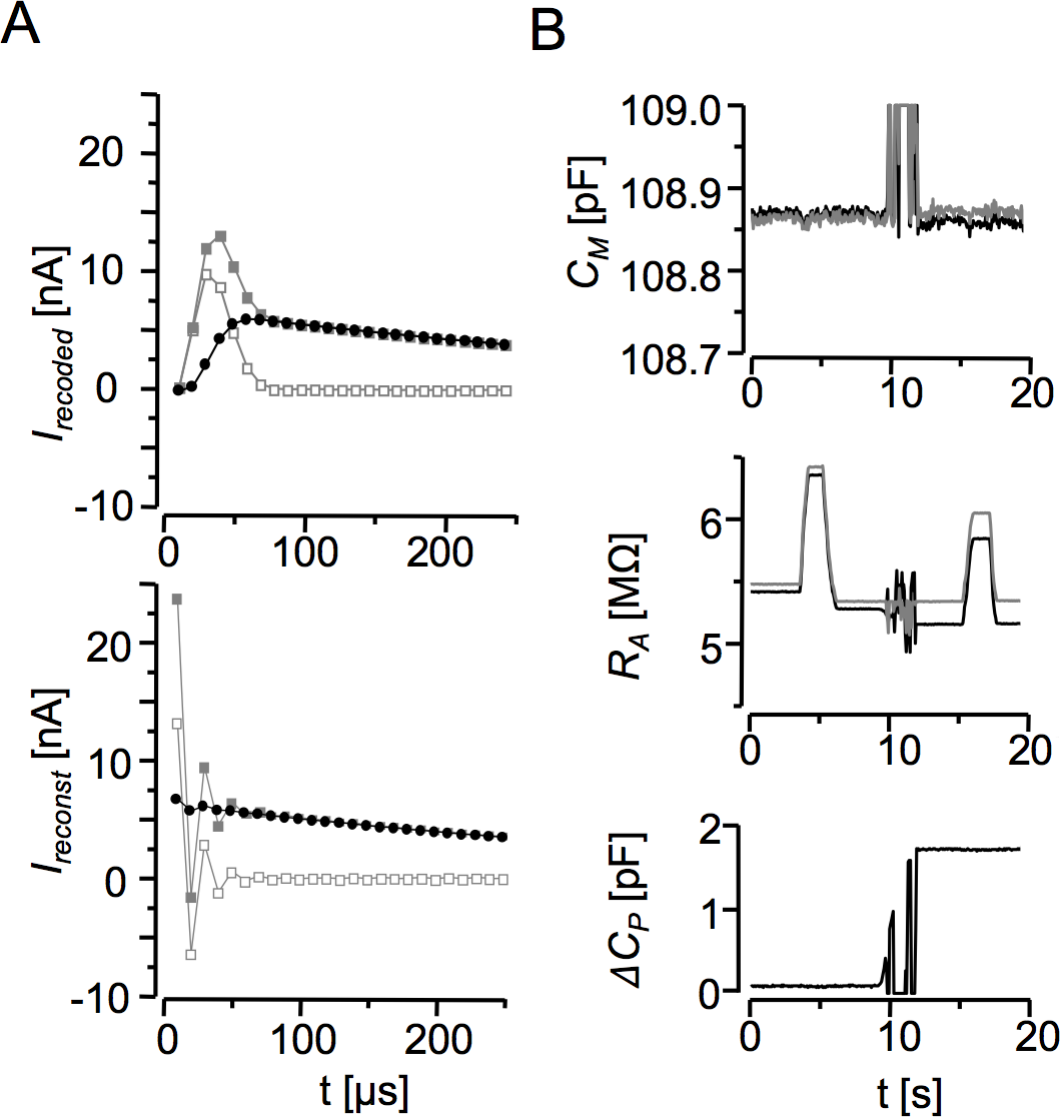
The effect of parasitic capacitance on estimates of impedance parameters. **A:** The onset of filtered current responses to step voltage changes with input of the measuring amplifier open and shielded. The compensation circuitry of the measuring amplifier was set to emulate cell circuit parameters. *Upper panel*: ●–the current response recorded with *R*_*A*_ set to 5.5 MΩ, *C*_*M*_ set to 109 pF, and the input capacitance well compensated. **□**–the current response recorded with decompensated input capacitance and *R*_*A*_ and *C*_*M*_ controls set to zero. ■–the combined current response recorded with decompensated input capacitance, *R*_*A*_ set to 5.5 MΩ, and *C*_*M*_ set to 109 pF. *Lower panel*: the current responses corresponding to records in the upper panel reconstructed by the deconvolution procedure. **B:** Effects of changes of parasitic capacitance (lower panel) on estimates of *C*_*M*_ and *R*_*A*_ of a cell emulated with the compensation circuitry of recording amplifier. Black traces–the time series of parameter estimates of current responses reconstructed by the deconvolution procedure. Grey traces–the time series of parameter estimates of current responses reconstructed with the deconvolution procedure applied to current responses with the first 60 μs blanked (note the absence of *C*_*P*_ crosstalk in *C*_*M*_ trace). The flickering artefacts are caused by handling of the fast capacitance compensation control. The change of parasitic capacitance Δ*C*_*P*_ was evaluated from the integral of the onset of recorded current response [10].

The parasitic capacitance, or stray capacitance, of a recording microelectrode, *C*_*P*_, connects to ground in parallel to cell membrane (Fig 1). It is charged very fast since it is not attenuated by the access resistance (Fig 5A, upper panel). The parasitic charging current is also reconstructed by the deconvolution procedure. Due to Gibbs phenomenon [3], the presence of *I*_*Cp*_ appears as dumped oscillation at the onset of the reconstructed current response.

To assess the problem of fast parasitic capacitance charging current (*I*_*Cp*_) for the deconvolution procedure, we emulated behaviour of the circuit with parasitic capacitance using compensation circuitry of the recording amplifier (Fig 5B). The relatively large increases in *C*_*P*_ setting by 1.8 pF (Fig 5B, lower panel) caused a small difference in both the *C*_*M*_ and *R*_*A*_ estimates from current records processed by the deconvolution procedure (Fig 5B, the upper and middle panel, respectively, black traces). The *C*_*P*_ dependent error could be excluded when the deconvolution procedure was applied to the current responses with the first 60 μs blanked to omit the oscillations in the reconstructed currents (Fig 5B, upper and middle panel, grey traces). Comparison of black and grey traces in Fig 5B indicates that partially uncompensated *C*_*P*_ affects the estimates of cell impedance parameters minimally. However, if *C*_*P*_ varied in time it would cause small artefacts in *C*_*M*_ and *R*_*A*_ traces, which look similar as if there was crosstalk of *R*_*A*_ to *C*_*M*_. Since we showed that crosstalk is not present in the case of the deconvolution procedure (cf. Fig 4), appearance of small correlated events in *C*_*M*_ and *R*_*A*_ traces indicates in real experiments instabilities in parasitic charging current. In these tests, the access resistance setting was shortly changed at the two *C*_*P*_ levels (Fig 5B, middle panel). As in previous tests, the crosstalk of *R*_*A*_ to *C*_*M*_ records was below the resolution of the *C*_*M*_ measurement, indicating that the oscillations in the reconstructed current due to uncompensated parasitic capacitance have negligible effect on *R*_*A*_ crosstalk to *C*_*M*_.

To overcome *C*_*P*_ interference, the current response to step voltage should be recorded in cell-attached configuration and subtracted from the analysed whole-cell current records. This removes oscillations from the deconvolved current responses and leaves unperturbed membrane current responses even at highest frequencies of the available bandwidth. When the input capacitance have changed substantially during experiment, blanking of the first few samples of recorded current responses before the deconvolution analysis could be applied if necessary (Fig 5B, grey traces).

### Tests on real cells

The quality of the deconvolution procedure was tested on isolated cardiac myocytes routinely used in our lab. The high-resolution *C*_*M*_ measurements in these cells were difficult by previous methods [14] for their large size (typically above 100 pF), variable membrane resistance (ranging from 50 to 1000 MΩ), and unstable access resistance. Plasmalemma in these cells displays complex spatial network of radial invaginations (t-tubules), caveolae, and endo/exocytotic vesicles. Since secretion of natriuretic peptides was also reported in these cells [14], it could be expected that active membrane processes would be present as in other cell types.

**Fig 6:**
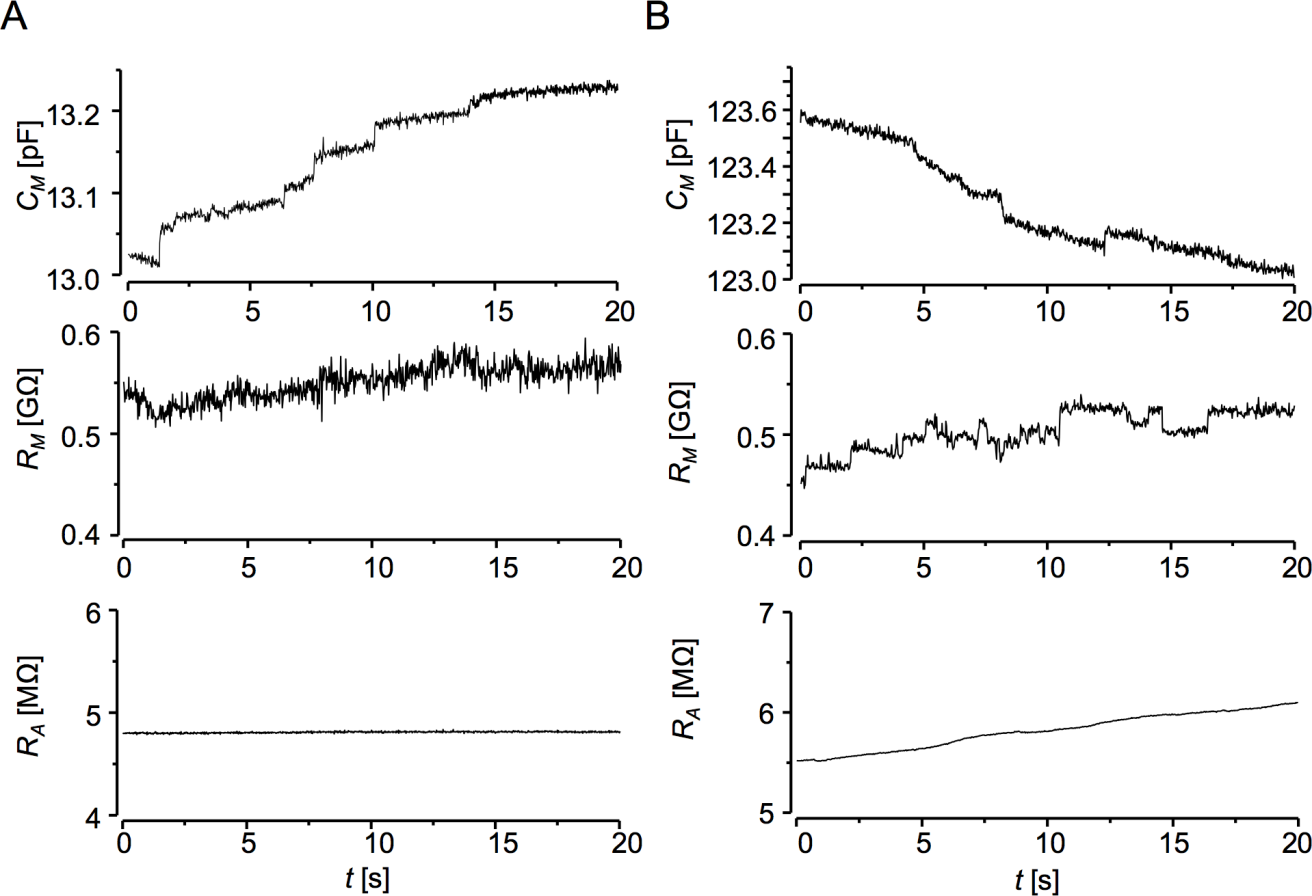
Tests of the deconvolution procedure on isolated cardiac myocytes. Note the absence of correlated changes in *C*_*M*_, *R*_*M*_ and *R*_*A*_ records indicating no visible cross-talk. **A:** Recording on a neonatal rat heart cardiomyocyte. Square-wave ±40 mV, 1.25 ms period, bandwidth 50 Hz. **B:** Recording on an adult rat heart cardiomyocyte. Square-wave ±40 mV, 10 ms period, bandwidth 50 Hz.

In Fig 6A, a small isolated cardiomyocyte of neonatal rat heart displays spontaneous stepwise increases in membrane capacitance of about 10 to 50 fF in amplitude without larger changes in membrane conductance and with a stable access resistance during the recording period. The theoretically expected noise in the *C*_*M*_ trace for 50 Hz bandwidth (Eq 3) was 1.0 fF, while the standard deviation of signal noise in the *C*_*M*_ trace was 2.6 fF, due to suboptimal period of voltage stimuli set to 1.25 ms, while for the charging time constant of 0.062 ms the optimal stimulation period would be 0.744 ms.

The recording on isolated cardiomyocyte of adult rat heart (Fig 6B) shows capacitive changes under less stable recording conditions when the access resistance increased gradually by 550 kΩ and the membrane conductance contained single channel-like activity of up to 80 pS conductance. Nevertheless, the deconvolution method neatly resolved stepwise capacitive events of 40 to 80 fF in the *C*_*M*_ trace without any cross-talk from *R*_*M*_ and *R*_*A*_ variation. At 50 Hz bandwidth, the standard deviation of signal noise in the *C*_*M*_ trace was 11.5 fF, while expected noise was 6.8 fF. The difference was partially due to suboptimal stimulation period (10 ms vs. 8.12 ms) and increased contribution of 1/f noise. The slow overall decrease in the *C*_*M*_ trace by 0.5 pF was due to relaxation of the intramembrane charge related to gating of voltage sensitive ion channels after changing the holding potential from -50 to 0 mV [7].

**Fig 7:**
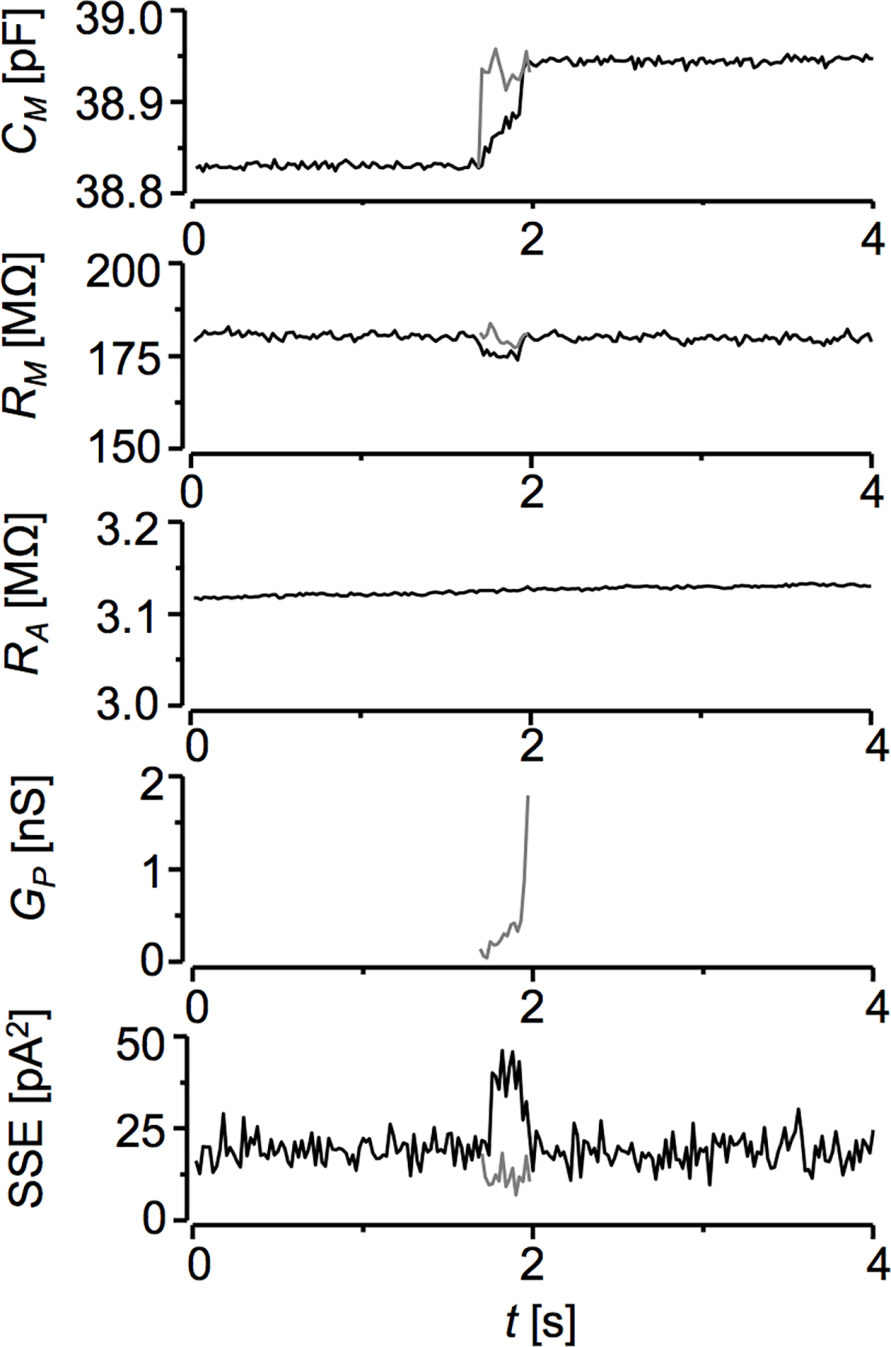
Detection of the fusion pore. A segment of current responses displaying about 100 fF membrane capacitance increase was analysed either by the three-element model (Eq 1, black traces) or with the five-element model (Eq 7, grey traces) of the input impedance. SSE—error of the fit of individual current responses by respective models. Isolated neonatal rat ventricular myocyte, holding potential 0 mV, square-wave ±40 mV, 1.25 ms period, bandwidth 50 Hz.

In Fig 7 we document the ability of the deconvolution method to resolve membrane capacitance events connected with prolonged opening of a fusion pore [5]. The capacitance event started with a gradual, not stepwise, increase (1.7 s of the trace time). At the same time, *R*_*M*_ trace showed a small negatively correlated change, despite the fact that the deconvolution procedure is free of cross-talk errors. Together with the accompanying increased error of the fit (SSE) of current responses (Fig 7, bottom panel) this indicated that the three-element impedance model (Eq 1) was inappropriate for description of the current. We employed Eq 7 as a five-element model for vesicle fusion [5].

$$I_{M}\left(t\right)=I_{S}+I_{P}\cdot e^{\left(\frac{-t}{\tau }\right)}+I_{P2}\cdot e^{\left(\frac{-t}{\tau 2}\right)},$$ (7)

where *τ2* = *C*_*v*_/*G*_*p*_, *C*_*v*_ is the capacitance of the fusing vesicle equal to the amplitude of the capacitance increase, *G*_*p*_ is the conductance of the fusion pore, and *I*_*P2*_ is the increase of the peak current amplitude due to fusion event (see [4] for details of the fitting procedure). The estimated fusion pore conductance increased from 0.1 to 0.5 nS within 250 ms and then jumped to an incalculable value, indicating that the pore opened wide and the fusion process had been completed.

### Seal resistance variation

**Fig 8:**
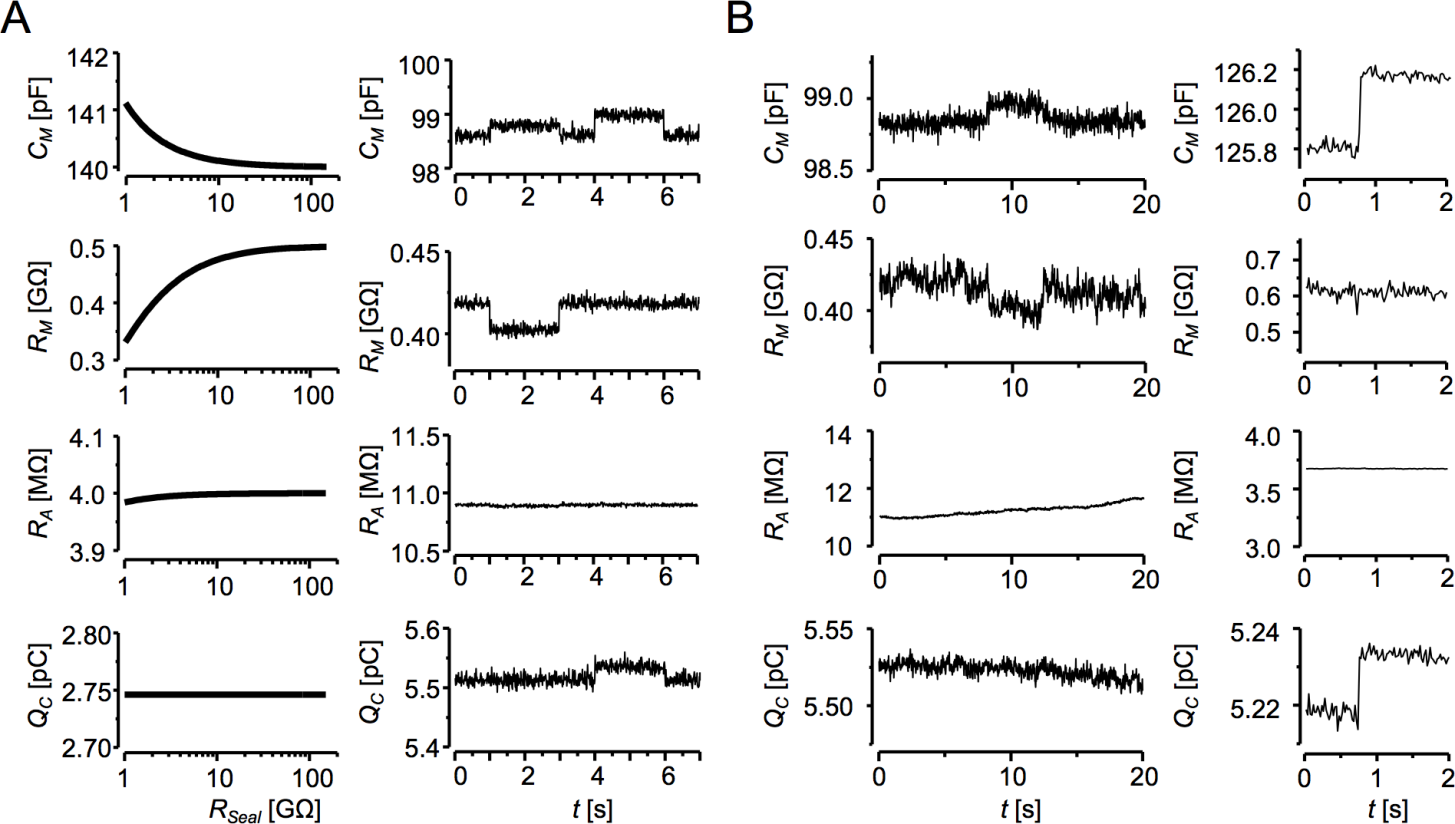
Effect of the seal resistance on circuit parameters. **A:** The simulated data. *Left panel*: Dependence of estimates of cell impedance parameters on *R*_*Seal*_ value (estimated by Eqs 1 and 8 from simulated current responses of the equivalent circuit according Eq 5, Fig 1A, *C*_*M*_ = 140 fF, *R*_*M*_ = 0.5 GΩ, *R*_*A*_ = 4 MΩ). *Right panel*: Simulated experiment with two events. The first event contained a step decrease of *R*_*Seal*_ from 100 to 10 GΩ while the second event contained a step change of *C*_*M*_ from 98.7 fF to 99.0 fF. Note that in the first event an anti-correlated step change emerged in *C*_*M*_ and *R*_*M*_ but not in *R*_*A*_ or *Q*_*C*_ traces, while in the second event a correlated change was present in the *C*_*M*_ and *Q*_*C*_ but not in *R*_*M*_ and *R*_*A*_ traces. The same set of circuit parameters as in the left panel, square-wave ±30 mV, 20 ms period, analysed by the deconvolution procedure. **B:** Records of impedance parameters of ventricular myocytes obtained by the deconvolution procedure. *Left panel*: A segment of a record containing an anti-correlated step change in *C*_*M*_ and *R*_*M*_ but not in *R*_*A*_ or *Q*_*C*_ traces (square-wave ±30 mV, 20 ms period), indicating artefactual change in membrane capacitance arising from the change of *R*_*Seal*_. *Right panel*: A segment of a record containing a well-resolved step *C*_*M*_ event without correlated changes in either *R*_*M*_ or *R*_*A*_ but mirrored in *Q*_*C*_ (square-wave ±20 mV, 10 ms period), indicating a true change of membrane capacitance.

There is a well-known but also well-hidden problem of the seal stability common to all patch-clamp measurements, since a stable and well isolating seal resistance is needed to confine the circuit current to the cell membrane (Fig 1A). The seal resistance is difficult to measure [15] but its quality can be assessed indirectly [12, 16]. If the cell membrane resistance reaches the gigaohm range, a small variation of *R*_*Seal*_ may cause the recorded current to be at variance with the membrane current. Thus, changes in *R*_*Seal*_ may affect estimates of current parameters in impedance measurements and generate artefactual events in current records. We tested the effect of *R*_*Seal*_ by simulation of the whole circuit (Fig 1A) using Eq 5. As evidenced in Fig 8A (left panel), reduction of *R*_*Seal*_ below 10 GΩ strongly affected estimates of *C*_*M*_ and *R*_*M*_ but had negligible effect on *R*_*A*_ estimates. Importantly, however, it had no effect at all on the charge under the capacitive membrane current response *Q*_*C*_, calculated as a simple integral of the current response above the steady current level according to Eq 8.

$$Q_{C}=\sum_{1}^{n}\left[I_{M}\left(n\right)-I_{S}\right]$$ (8)

The illustrative experiment on a cardiomyocyte (Fig 8B) shows an upsurge of *C*_*M*_ that correlated with the drop in *R*_*M*_ but not in *R*_*A*_ and *Q*_*C*_ records. Since the deconvolution-based procedure is effectively free of the cross-talk error, the observed combination of events indicates that this change did not happen at the cell membrane and should be considered as an artefact caused by variation of *R*_*Seal*_. Indeed, in the case of true *C*_*M*_ change, as shown in Fig 8B, the large event in the *C*_*M*_ record was not correlated with *R*_*M*_ or *R*_*A*_ but was reflected in *Q*_*C*_.

The charge *Q*_*C*_ was unaffected by *R*_*Seal*_ as expected, because only *I*_*M*_ but not *V*_*M*_ is affected by *R*_*Seal*_. This trait is a precious signature of an unstable seal. Independence of *Q*_*C*_ on step change of *R*_*Seal*_ was confirmed in a simulated experiment (Fig 8A, right panel). A step change of *R*_*Seal*_ caused artefactual increase of *C*_*M*_, decrease of *R*_*M*_, and no change of *R*_*A*_, in their respective traces. For comparison, a true increase of *C*_*M*_ caused no change of *R*_*M*_ and *R*_*A*_ but caused a correlated increase in *Q*_*C*_.

## Discussion

Precise recording of cell membrane currents is essential for exact characterization and correct interpretation of membrane phenomena related to electrical processes. In this study, we described the deconvolution procedure for reconstruction of the high frequency content of membrane current distorted by the recording system. The deconvolution procedure was tested by measurements of current responses to square voltage pulses using the software and hardware means, including experiments on real cells. Since the membrane charging response is very fast, these measurements need high fidelity recordings. On the other hand, the theory of capacitance charging is simple and allows exact testing. We showed that the deconvolution procedure applied to recorded currents recovered their theoretical time course expected for the available bandwidth at the input of the recording amplifier.

The deconvolution procedure primarily solved the problem of membrane current distortion by the antialiasing low-pass filter at the output of the recording system. However, by doing this it also improved the bandwidth, signal to noise ratio, and high-frequency information content of digitized current records. Generally, this allows better characterization of faster processes and their exact interpretation.

In this study, we presented results obtained with membrane currents recorded at 10 kHz low-pass filtering and 100 kHz digitization. The question was, whether an increased bandwidth would further improve the accuracy and resolution of the measurements. To this end, we tested the deconvolution procedure with the low-pass filter set to 100 kHz and the digitizer set to 250 kHz. The test measurements on hardware cells did not confirm the expectations, because the noise in current records was substantially increased. Moreover, the much larger volume of data increased the demands on computational time. On the contrary, the deconvolution procedure worked very well with 5, 2, and 1 kHz low-pass filtering combined with 50 or 10 kHz digitization, at the corresponding bandwidth of 25 or 5 kHz, respectively (not shown).

We showed that the deconvolution procedure substantially improved performance of the membrane capacitance measurements by increase of the absolute accuracy and by suppression of the crosstalk error among the impedance parameters. As a result, the method detects minute membrane capacitance changes that can be interpreted with confidence and even semi-automatically [17]. The whole deconvolution and analysis procedure, as implemented in the MAT-MECAS software (http://mat-mecas.sourceforge.net), works well with cells of diverse membrane capacitance and with common patch-clamp setups in a wide range of experimental conditions and designs.

The square-wave method of membrane capacitance measurement suffers as any high-resolution method from three critical issues common to all patch-clamp based approaches; namely, the parasitic capacitance, the seal resistance, and the access resistance. These should be fairly stable for reliable performance.

The uncompensated *C*_*P*_ gives rise to large and fast charging currents that cause excessive ringing of the output filter. After deconvolution, the ringing due to *C*_*P*_ charging is much briefer and ends after tens not hundreds of microseconds. Uncompensated *C*_*P*_ might introduce a small error in the estimate of *R*_*A*_. However, due to eliminated crosstalk errors, changes of *C*_*P*_ below 1 pF will not have visible influence on *C*_*M*_ records. The filter ringing caused by somewhat slower cell capacitance charging current was suppressed by the deconvolution procedure to the extent that would not interfere with fast and miniscule processes like membrane charge movement.

We showed that the seal resistance above 10 GΩ would be safe for artefact-free recordings in most situations. If the seal loses its stability and drops to the low gigaohm range, it could be recognized in impedance measurements as an anti-correlated change in *C*_*M*_ and *R*_*M*_ traces with no change in *R*_*A*_ and *Q*_*C*_ traces. To our experience with cardiac myocytes, the issue of the seal resistance can be overcome by thorough formation of the gigaseal contact [15,16].

The deconvolution method is insensitive to variation of the access resistance in contrast to other method [4,5,6]; nevertheless, the *R*_*A*_ value determines the time constant of recording. Therefore, in experiments with high-resolution membrane capacitance recording the optimal period of the stimulation waveform should be considered for the best *C*_*M*_ resolution [5]. Faster stimulation rates provide higher resolution [18]; at the same time, a shorter square-wave period provides less data points for fitting, what increases the impact of the noise in recorded current and decreases accuracy of the fit. Therefore, the decision regarding optimal patch pipette resistance for a given cell size is a trade-off between resolution and accuracy.

In our experiments on cardiac myocytes, the variable membrane conductance did not cross-talk to membrane capacitance records, as expected for the square wave method. However, it contributed to the noise in *C*_*M*_ records through increased error of the fit to noisier current responses. Still, due to the deconvolution procedure, we could reliably resolve capacitive changes above few fF or the conductance of the fusion pore. Here we show records of spontaneous membrane activity, which may be related to development of plasmalemma [19] and/or intracellular calcium signals [20]. It should not be forgotten, that the membrane impedance may react also to changes in the physical state of the cell membrane by changes in dielectric constant, charge redistribution, or membrane permeability. Nevertheless, the changes in the membrane capacitance due to physical factors are smooth and gradual. The voltage dependent component may account for up to about 15% of the overall membrane capacitance [7], while the change of bath temperature by 1°C may change membrane capacitance by about 0.3% [21].

In conclusions, the deconvolution procedure increased the bandwidth of the recorded current responses; however, the essence of its unique contribution is in correct restoration of the time course of current responses. Thus in recordings of membrane current it brought compliance with theoretical expectations. In the case of mono-exponential current decay, approximation of the reconstructed current response by the theoretical equation became correct and robust for a wide span of current and membrane parameters. The minimal time constant of the membrane current decline that returned reliable results was 20 μs, corresponding to a cell of 2 pF capacitance (8 μm in diameter) measured with 10 MΩ access resistance.

## References

[1] Sakmann B. and Neher E. Single-channel recording. Plenum Press, NY, 1983.

[2] Sigworth FJ. Electronic design of the Patch Clamp In: Sakmann B. and Neher E. (eds.), Single-channel recording. Plenum Press, NY, 1983.

[3] Smith SW. The scientist and engineer's guide to digital signal processing. (California Technical Publishing, San Diego, CA, USA, 1997. ISBN:0-9660176-3-3)

[4] Lindau M, Neher E. Patch-clamp techniques for time-resolved capacitance measurements in single cells. Pflugers Arch. 1988;411:137–146. 3357753 10.1007/BF00582306

[5] Thompson RE, Lindau M, Webb WW. Robust, high-resolution, whole cell patch-clamp capacitance measurements using square wave stimulation. Biophys J. 2001;81:937–948. doi: 10.1016/S0006-3495(01)75752-9 11463636 10.1016/S0006-3495(01)75752-9 PMC1301564

[6] Gillis KD. Techniques for membrane capacitance measurements In Single-Channel Recording, 2nd ed. Sakmann B., and Neher E. (editors). Plenum Press, NY, 1995.

[7] Novák P, Zahradník I. Q-method for high-resolution, whole-cell patch-clamp impedance measurements using square wave stimulation. Ann Biomed Eng. 2006;34:1201–1212. doi: 10.1007/s10439-006-9140-6 16786392 10.1007/s10439-006-9140-6

[8] Rohlicek V, Schmid A. Dual-frequency method for synchronous measurement of cell capacitance, membrane conductance and access resistance on single cells. Pflugers Arch. 1994;428:30–38. 7971159 10.1007/BF00374749

[9] Gillis KD. Admittance-based measurement of membrane capacitance using the EPC-9 patch-clamp amplifier. Pflugers Arch. 2000;439:655–664. 10764227 10.1007/s004249900173

[10] Santos-Sacchi J. Determination of Cell Capacitance Using the Exact Empirical Solution of ∂Y/∂Cm and Its Phase Angle. Biophys J. 2004;87:714–72. doi: 10.1529/biophysj.103.033993 15240504 10.1529/biophysj.103.033993 PMC1304394

[11] Lindau M. High resolution electrophysiological techniques for the study of calcium-activated exocytosis. Biochim Biophys Acta 2012;1820:1234–1242. doi: 10.1016/j.bbagen.2011.12.011 22209782 10.1016/j.bbagen.2011.12.011 PMC3323678

[12] Mason MJ, Simpson AK, Mahaut-Smith MP., Robinson HPC. The Interpretation of Current-Clamp Recordings in the Cell-Attached Patch-Clamp Configuration. Biophys J. 2005;88:739–750. doi: 10.1529/biophysj.104.049866 15516522 10.1529/biophysj.104.049866 PMC1305049

[13] Janíček R, Zahradníková A Jr, Poláková E, Pavelková J, Zahradník I, Zahradníková A. Calcium spike variability in cardiac myocytes results from activation of small cohorts of ryanodine receptor 2 channels. J Physiol. 2012;590:5091–5106. doi: 10.1113/jphysiol.2012.234823 22890710 10.1113/jphysiol.2012.234823 PMC3497565

[14] Ryu SY, Lee SH, Isenberg G, Ho W, Earm YE. Monitoring of ANP secretion from single atrial myocytes using densitometry. Pflugers Arch. 2002;444:568–577. doi: 10.1007/s00424-002-0852-7 12136277 10.1007/s00424-002-0852-7

[15] Fischmeister R, Ayer RK Jr., DeHaan RL. Some limitations of the cell-attached patch clamp technique: a two-electrode analysis. Pflugers Arch. 1986;406:73–82. 2419832 10.1007/BF00582957

[16] Perkins KL. Cell-attached voltage-clamp and current-clamp recording and stimulation techniques in brain slices. J Neurosci Methods. 2006;154:1–18. doi: 10.1016/j.jneumeth.2006.02.010 16554092 10.1016/j.jneumeth.2006.02.010 PMC2373773

[17] Hoťka M, Zahradník I. Low-crosstalk whole-cell membrane capacitance recording method. Biophys J. 2016;110(3):p429a.

[18] Chen P, Gillis KD. The noise of membrane capacitance measurements in the whole-cell recording configuration. Biophys J. 2000;79:2162–2170. doi: 10.1016/S0006-3495(00)76464-2 11023920 10.1016/S0006-3495(00)76464-2 PMC1301106

[19] Macková K, Zahradníková A Jr, Hoťka M, Hoffmannová B, Zahradník I, Zahradníková A. Calcium release-dependent inactivation precedes formation of the tubular system in developing rat cardiac myocytes. Eur Biophys J. 2017; doi: 10.1007/s00249-017-1249-z 28913625 10.1007/s00249-017-1249-z

[20] Zahradník I, Hotka M. Membrane capacitance changes in isolated rat cardiac myocytes. Acta Physiologica 2016;217(S708):p44.

[21] Hotka M, Zahradnik I. Membrane capacitance changes due to temperature increase in rat cardiac myocytes. Biophys J., 2014; 106(2):p121a–122a.

